# Oral lichen planus: a novel staging and algorithmic approach and all that is essential to know

**DOI:** 10.12688/f1000research.18713.1

**Published:** 2020-03-24

**Authors:** Eran Shavit, Hagen Klieb, Neil H. Shear

**Affiliations:** 1 Division of Dermatology, Department of Medicine, Sunnybrook Health Sciences Centre, University of Toronto, Toronto, ON, Canada; 2 Dermatology Unit, Barzilai University Medical Center, Ashkelon, Israel; 3 Faculty of Health Sciences, Ben-Gurion University of the Negev, Beer-Sheba, Israel; 4 Department of Dentistry, Sunnybrook Health Sciences Centre, University of Toronto, Toronto, ON, Canada; 5 Department of Anatomic Pathology, Sunnybrook Health Sciences Centre, University of Toronto, Toronto, ON, Canada; 6 Department of Medicine (Dermatology, Clinical Pharmacology, and Toxicology), Sunnybrook Health Sciences Centre, University of Toronto, Toronto, Ontario, Canada; 7 Department of Pharmacology, Sunnybrook Health Sciences Centre, University of Toronto, Toronto, ON, Canada

**Keywords:** lichen planus, oral lichen planus, erosive oral lichen planus, atrophic oral lichen planus

## Abstract

Lichen planus (LP) is a chronic autoimmune disease. Oral lesions may occur in isolation or in combination with other affected muco-cutaneous sites. Oral LP (OLP) may present as one of the various manifestations of LP or may present as a disease sui generis with a broad range of severity. Despite this disease targeting the oral mucosa, its great impact on quality of life is underrated. In this article, we provide an updated review of the pathophysiology and epidemiology of OLP and offer guidance for its diagnosis and management. We also propose an algorithmic approach to the clinical forms of OLP and a novel staging system to facilitate management decisions.

## Introduction

Lichen planus (LP) is a chronic inflammatory disease that affects the skin, hair, nails, and mucous membrane including the oral cavity. It usually presents with recurrences and periods of clinical exacerbations and remissions; however, it may be a transient disorder ^1^. Oral LP (OLP) is a common disease that afflicts 1–2% of the population, most commonly middle-aged females, and is persistent ^2^. A chronic course is typical, and spontaneous resolution is uncommon ^3, 4^. OLP may take a progressing course and therefore require lifetime surveillance. Although LP is managed medically by dermatologists, OLP is mostly encountered and managed by oral surgeons. The aim of this review was to summarize the essentials required to understand OLP to facilitate its diagnosis and management and to encourage a multidisciplinary approach.

Our literature search was performed using PubMed with keywords such as “oral lichen planus” and/or “oral lichen planus” with other keywords to extend the search such as “etiology”, “epidemiology”, “diagnosis”, “management”, and “therapy”. Excluded from our review were articles that did not specifically adhere to OLP, such as oral lichenoid reactions, or articles that include other nonspecific stomatitis indicated in the article title, and articles published in languages other than English. Generally, no restrictions were made on any article type, but clinical trials and reviews were our primary focus. Additionally, some individual articles retrieved manually from the reference list of the relevant papers were also included.

The aim of this article is to review the etiopathogenesis, diagnosis, and treatment of OLP and to propose a novel algorithm and staging approaches to facilitate the work of clinicians who may encounter them in their practice.

## Background on OLP

OLP is a common disease encountered worldwide, with a female preponderance ^2– 4^. The overall incidence of OLP is uncertain but is estimated to be between 0.5 and 1.5% ^5^. The involvement of the oral mucosa in systemic LP patients is between 70 and 77% ^6^. The disease commonly presents in the older population, between the fifth and sixth decade of life. This disease is uncommonly seen in younger patients. This was supported in a recent study investigating oral mucosal lesions in teenagers: only 0.13% out of more than 6,000 patients actually suffered from OLP as a cause of mucosal lesion ^7^. The disease is influenced by local aggravating factors including tobacco smoking, dry mouth, mechanical irritants, and bacterial plaque. Despite the latter-mentioned aggravating factor, patients should be reassured that OLP is not an infectious disease.

The etiology of OLP is still unknown. Also, the pathogenesis is not entirely clear, but it is thought to arise from an immune response presumably involving CD4 ^+^ and CD8 ^+^ T lymphocytes producing cytokines, interleukin-2, and tumor necrosis factor within the oral epithelium that induce a chronic inflammatory response and keratinocyte apoptosis ^8^. Hence, there is no surprise that this disease has also been associated with other illnesses that share an immunological basis ^9^. OLP is seen in families, which implies that genetic predisposition exists for this disease ^10, 11^. Indeed, genetic polymorphisms of various human leukocyte antigen (HLA) markers have been suggested; gene polymorphisms of different HLA markers, as well as inflammatory cytokines and chemokines, have been associated with the presence of LP. Although the cause of these polymorphisms is not clear, their occurrence supports the autoantigen hypothesis ^12^.

Interestingly, the association with other autoimmune diseases, not restricted to OLP, has been recently rationalized with the discovery of ‘Survivin’, a multifunctional protein that is part of the inhibitor of apoptosis family and is functionally important for cell division, apoptosis, and, possibly, microRNA biogenesis. This protein has been found to be involved in various autoimmune diseases, hence shedding more light on their pathogenesis ^13^.

The great impact OLP has on its patients is so tremendous that one study examining 50 patients with OLP compared with controls showed a greater degree of sleep problems in such a way that their sleep deprivation has led to psychiatric disorders such as depression and anxiety ^14^. Another study showed that the longer the duration of subjective symptoms, the poorer the quality of life and the higher the level of perceived stress ^15, 16^. These findings were further backed up in a few recent reviews, including one from the past year, that all indicate that the development of OLP and especially the exacerbation of this disease is linked with stress, anxiety, depression, and eventually impairment of the patient’s quality of life ^17– 19^. The rationale behind this was proved with elevated salivary and/or urinary cortisol levels that corresponded to increased anxiety and depressive states ^19^.

## Comorbid risks with OLP

LP with oral involvement has been linked to other systemic diseases with different levels of evidence and various results. The strongest association exists with hepatitis C virus (HCV) ^20^. Although HCV has been strongly linked to systemic LP, not many studies have focused solely on OLP ^21^. Also, the coexistence of HCV infection and OLP may be relevant in some geographic regions such as southeast Asia and southern Europe ^22^ but less relevant in other regions such as central Europe, as it was exhibited in another recent study ^23^. LP has also been said to be affected by some other viruses such as those of the herpes family, but, in contrast to the latter, this was not substantiated with OLP ^24^. However, a much more significant association has been determined recently between human papilloma virus (HPV) and OLP; this association, as with HCV, also varies with different geographic populations. This association suggests a causal role in the malignant progression of OLP, which is of great significance, since OLP is benign in nature, but with this association malignant transformation is a potential risk, although it may not apply in all cases ^25^. Other associations that are mostly related to LP and not OLP in the strict sense include diabetes mellitus, hypertension, hypercholesterolemia, and rarely thymoma ^9, 21, 22, 26^. Good’s syndrome is another example of a disease that has an association with OLP but an even rarer occurrence when thymoma is combined with hypogammaglobulinemia ^26^.

Autoimmune diseases were present in 7% of OLP patients in one study but were not statistically significant when compared to a control group ^22^.

Only a few studies showed a true association between OLP and other systemic diseases, of which only a few were controlled studies with a high level of evidence ^27^ (see Table 1 for a summary of studies associated with systemic diseases). However, equal or more studies have depicted results that disprove these associations, and indeed most of these associations are based on cases series and retrospective studies with relatively small samples studied ^28– 32^.

**Table 1.** Summary of previous studies supporting the association of OLP with other diseases since 2000 ^2, 9, 25, 28, 36, 37^.

Author/journal/year	PubMed ID	Country	Sample size	Systemic disease	Study design and methods	Outcome
Ma J, *PLoS One*. 2016 ^25^	27571417	China	835 cases and 734 controls	HPV	Meta-analysis	HPV 16 and 18 showed strong association with OLP
Lauritano *et al*. *Head* *Face Med*. 2016 ^9^	27113338	Italy	87 OLP patients	HTN, DM, HCV thyroiditis	Retrospective chart review	Only one patient developed a malignant transformation (1.2%)
Barbosa *et al*., *Int J* *Dermatol*. 2015 ^36^	25534406	Brazil	37 OLP patients	HTN, DM Less HCV, anxiety	Case series	No significant association was observed between the variables studied and clinical form or Sx
Gümrü B. *Med Oral Patol* *Oral Cir Bucal*. 2013 ^28^	23524413	Turkey	370 OLP patients	HTN, DM, anxiety & depression	Retrospective chart review	Multiple sites in the majority of patients No malignant transformation
Bermejo-Fenoll *et al*. *Oral* *Pathol Med*. 2010 ^37^	20456611	Spain	550 OLP patients	HTN, rheumatic diseases, GI disorders, anxiety & depression	A retrospective descriptive study	Five patients (0.9%) developed SCC
Eisen. *J Am Acad* *Dermatol*. 2002 ^2^	11807431	USA	723 OLP patients	HCV	Retrospective study	Oral SCC developed in six patients(0.8%)

DM, diabetes mellitus; HCV, hepatitis C virus; HPV, human papilloma virus; HTN, hypertension; GI, gastrointestinal; SCC, squamous cell carcinoma; Sx, symptoms.

## Differential diagnosis and prognosis

The diagnosis of OLP begins with a thorough history and physical examination. Patients may or may not have symptoms. Although there is no consensus regarding the subtypes of OLP, commonly few variants present, including reticular, papular, plaque-like, atrophic, and ulcerative (erosive). The reticular form of OLP is typically asymptomatic and characterized by lacy or striated keratosis. Patients may be aware of an altered oral texture (usually described as a rough sensation similar to sandpaper). On occasion, there will be irritation with hot and acidic foods or beverages. There can be chronic and lingering oral burning discomfort with erythematous and ulcerative lesions. Although experienced dentists and dermatologists can often diagnose OLP clinically, a biopsy may be necessary to exclude immunobullous disorders (e.g. mucous membrane pemphigoid and pemphigus vulgaris) if there is extensive ulceration and mucosal desquamation. Biopsy is essential and must also be considered if there is concern regarding dysplastic or neoplastic transformation ^32^, for example, a region of persistent and progressive erythema, induration, or tissue friability. Moreover, persistent symptomatic or asymptomatic red and white lesions in the oral cavity should never be ignored; these oral lesions must be biopsied. Patients complaining of dysphagia or odynophagia should be referred to a gastroenterologist to exclude esophageal LP with endoscopy.

OLP must be distinguished from other lichenoid lesions, including lichenoid drug reactions, lichenoid amalgam reactions, and graft-versus-host disease. Signs and symptoms of these disorders mimic OLP, and biopsy will be of limited value since the histopathological features are similar to OLP, but direct immunofluorescence is of importance to confirm the diagnosis. Nevertheless, a thorough medical history will usually facilitate diagnosis ^33^. Although currently there is no blood test to determine the diagnosis of OLP, blood tests may be required to rule out the presence of associated comorbidities such as diabetes mellitus, dyslipidemia, thyroid diseases, and chronic liver diseases.

## Histopathology of OLP

The histological features of OLP include epithelial hyperkeratosis (orthokeratosis or parakeratosis), liquefactive degeneration of the basement membrane, and classically a band-like lymphocytic infiltrate in the superficial lamina propria comprising colloid or civatte bodies, which are degenerated keratinocytes in the interface of the epithelium and mucosa. A saw-tooth pattern of the epithelial ridges is more commonly expected in the dermis of LP rather than in the mucosa of OLP ^33– 35^. Histopathological evaluation is essential to precisely determine the diagnosis of OLP versus other diagnoses but more often to rule out the possibility of neoplastic transformation that is included in the differential diagnosis but also may be merely a complication of longstanding OLP.

## Current classification of OLP

**Figure 1.**
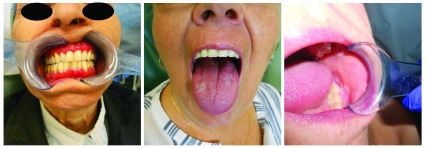
Images of patients with desquamative gingivitis, a leukoplakia like oral lichen planus, and reticular type oral lichen planus. We confirm that we have obtained written consent to use these images from the patients included in this figure.

There are a few variants of OLP, including atrophic, erosive, bullous plaque-like, and reticular forms. However, these variants are often intermingled. The reticular form presents with characteristic lacy white striae with a diffuse and bilateral distribution in the oral mucosa commonly affecting the buccal mucosa, ventral tongue, and gingiva. The atrophic and erosive forms present as red patches and ulceration, respectively. Plaque-form LP resembles leukoplakia but has a multifocal distribution. The bullous variant is rare, with bullae or vesicles that can resemble other oral immunobullous disorders (see Figure 1 for various types of OLP).

**Figure 2.**
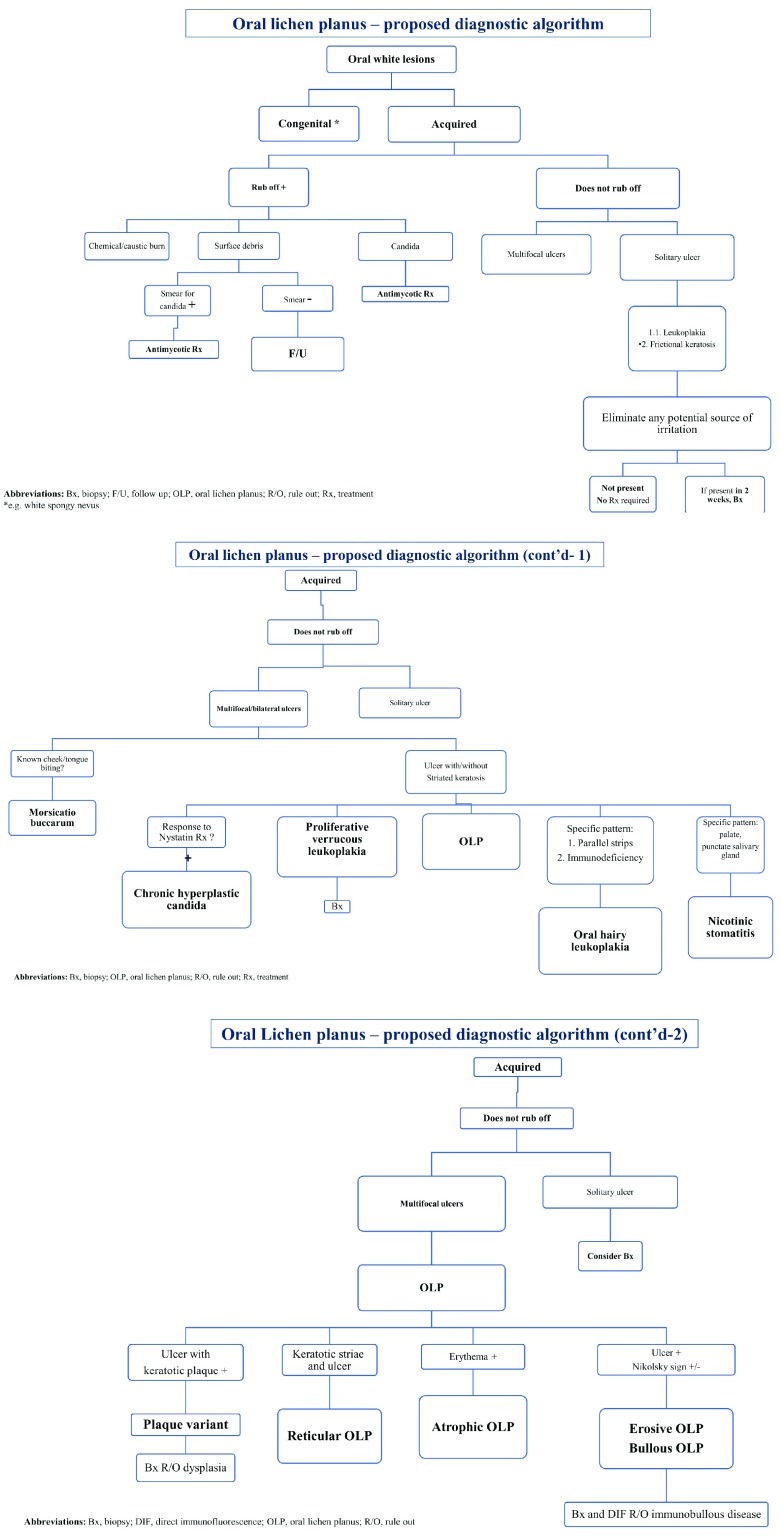
Oral lichen planus – proposed diagnostic algorithm. Bx, biopsy; DIF, direct immunofluorescence; F/U, follow up; OLP, oral lichen planus; R/O, rule out; Rx, treatment; *e.g. white spongy nevus

Currently, there is no consensus regarding the classification of OLP, and in various reports the disease is characterized based on the different clinical forms and/or whether the disease is symptomatic or not. We believe that this gap should be better addressed in order to facilitate the work of clinicians. For the sake of simplicity, one approach is to categorize the OLP based on the degree of involvement as mild, moderate, or severe. However, since there are many subjective complaints, i.e. the mild cases may be described as severe in certain individuals, these complaints may and should dictate management. Another option is to divide the disease according to whether or not symptoms are available, namely symptomatic or asymptomatic. But this is not an adequate approach that will differentiate the different variants of this disease. For instance, whereas the reticular variant may be typically asymptomatic, the other variants may manifest with variable burning discomfort that is exacerbated by certain acidic and tart foods and beverages as well as with toothpastes and mouthwashes. There can be severe lingering pain affecting the patient’s quality of life but little or no impact when the disease is asymptomatic in one study ^38^. For the clinical approach to various forms of OLP, see Figure 2.

Forming an algorithmic approach is essential, since it is not always possible to generalize and to decide on management based solely on the clinical morphology. Nevertheless, we sought to suggest an algorithmic approach to facilitate the diagnosis of OLP (see Figure 2). It is essential to emphasize that our novel algorithm is only a suggested one and should be tested before widely accepted. Also, we would like to suggest a novel classification system for this disease in which it is separated into three stages—I, II, and III—that will correspond to, but better depict, three levels of severity—mild, moderate, and severe, respectively (see Table 2). Determining the level of involvement is pivotal for establishing the management strategy. Although it is a disease localized to one region, it should be considered as a systemic variant of LP. Moreover, a high prevalence of esophageal involvement was found in patients with systemic LP ^39^. In another recent study, 62% of the sample studied had esophageal involvement, of which 90% of patients with esophageal disease had oral involvement. However, that cohort was composed of a small sample ^40^.

**Table 2.** A new classification of OLP.

Stages of disease	Description	Management plan	Recommended discipline
I: mild	No symptoms	No pharmacotherapy indicated May use nonpharmaceutical substances	Dentist
II: moderate	Mild-to-moderate symptoms (e.g. sensitivity with spicy foods, mild lingering pain)	Low-potency corticosteroids/alternative TCIs, treat PRN	Preferably oral maxillofacial surgeon specialist and/or dermatologist
III: severe	Severe or recalcitrant symptoms/systemic manifestations and/or diffuse involvement of the entire oral cavity	First-line high-potency corticosteroids, systemic treatment (see text)	Dermatologist or another immunotherapy specialist May involve consultants as well such as ENT specialists

ENT, ear, nose, and throat; OLP, oral lichen planus; PRN, *pro re nata* (when necessary); TCI, topical calcineurin inhibitor.

## Complications of OLP

We believe that OLP is not only associated with other systemic diseases (as mentioned previously) but also a systemic disease by itself that presents regionally. Naturally, it is impacted by other diseases that may shorten the lifespan of patients. But other complications do exist, including infection and malignant transformation. Despite the wide use of topical corticosteroids to manage patients with OLP, studies have failed to show a statistically significant increase in the risk for oral candidiasis; however, clinicians should be aware of it and treat it when it appears rather than prophylactically ^41^.

The risk of malignant transformation is uncertain, but, although it exists, it is most probably much lower than once previously thought. Nevertheless, it was enough to include OLP in one of the oral malignant disorders ^42^. In one systematic review, the overall rate of OLP patients who eventually suffered malignant transformation to squamous cell carcinoma (SCC) was 1% ^43^. Another more recent study showed that patients with OLP were 4.8 times more likely to have oral SCC than the matched referents ^44^. In another Finnish population survey involving more than 13,000 LP patients, the risks of cancers of the oral mucosa, esophagus, and larynx were significantly elevated. The standardized incidence ratio for cancer of the tongue was 12.4 (95% confidence interval [CI] 9.45–16.0) and for cancer of the oral cavity was 7.97 (95% CI 6.79–9.24) ^45^ Two other recent systematic reviews have also showed a low risk of potential malignant transformation: one with an overall malignant transformation rate to SCC of 1.4% ^46^ and another with a combined malignant transformation rate to SCC of 1.14% ^47^. However, both studies have identified specific risk factors for malignant transformation to include tongue localization, atrophic-erosive lesions, tobacco smoking, and alcohol consumption ^46, 47^.

## Management

There is no consensus despite scarce reports about an algorithmic approach towards the management of OLP ^48^. In general, management should be directed towards symptoms. No therapy is advocated in the absence of symptoms. Irritating foods, beverages, and oral hygiene products (e.g. minty toothpastes) should be avoided. Optimum oral hygiene and regular dental cleanings are helpful for minimizing plaque and gingival inflammation with the potential to exacerbate this condition. Pharmacotherapy is indicated, however, when symptoms are severe, lingering, or interfering with daily functions (e.g. toothbrushing, eating). The rationale behind the therapy is based on further insight into the pathogenesis of OLP as an immune-mediated disease related to T-lymphocyte immunological dysfunction, and within the implicated cytokines, such as TNF-alpha, IFN-gamma, TNF-alpha, IL-6, and IL-8, which has paved the way for the utilization of immunosuppressant therapies. Therefore, commonly, topical corticosteroids are utilized initially for symptomatic OLP, and, if topical therapy fails, systemic therapy is considered.

However, owing to the chronic nature of this disease, a complete cure is very difficult to achieve. There is no uniform approach to treatment, with treatment varying from one individual to another. Nevertheless, in recalcitrant cases, calcineurin inhibitors (such as tacrolimus and pimecrolimus) have been introduced as a second line of therapy. These medications should be used by experts with caution, preferably for short-term treatments, owing to the risk of developing oral SCC after its usage ^49, 50^.

Only scarce clinical trials are available that examined the effect of systemic medications such as methotrexate, systemic pulse therapy of corticosteroids, alitretinoin, and, more recently, apremilast ^51, 52^. Studies on systemic therapies in the form of energy-based devices such as photodynamic therapy or laser therapy, or these in combination with corticosteroids, were also published ^53– 56^. However, many more reports advocate the utilization of topical medications, especially corticosteroids either alone or in combination in various chemical preparations ^57^. More than a dozen studies have investigated the use of topical calcineurin inhibitors (pimecrolimus or tacrolimus) either alone or compared to corticosteroids ^58, 59^. Also, other topical components such as cyclosporine have been examined ^60– 62^. Fewer attempts are made with various other substances such as *Aloe vera* gel or other “natural” substances or topical preparations such as tazarotene with various results in anecdotal reports ^63– 65^. What is the reason for more reports of various topical preparations versus systemic therapies? One explanation is that clinicians and patients are settling in the use of topical medications for OLP and reserving systemic medications for when the former fails. Another explanation is since there is no gold standard therapy nor consensus or guidelines on how to treat these patients, the management decision is being made on a case-by-case basis.

## Conclusions

OLP is a common disease with an overwhelmingly negative impact on the patient’s quality of life. The diagnosis is mostly based on clinical grounds, but it seems that histological and immunofluorescence confirmation is necessary owing to many reasons we have mentioned. Despite being localized only to the oral cavity, in our opinion, OLP should be recategorized as a systemic disease because of the major impact it has and should not be considered as merely one of the manifestations of LP. We do not know whether there is an immunological relationship among LP, OLP, etc., or if indeed this will help us define a therapeutic approach that aligns with the ideal target at each stage. Nevertheless, there is still no uniform approach towards the management of OLP and, except for the widely used topical corticosteroids, the therapy varies greatly between clinicians and medical institutions. This might stem from the lack of uniform classification. We have suggested an easier new classification system and algorithmic approach to the different variants of the disease. Our suggested algorithm should be critically appraised and tested in the future. However, we hope that this will facilitate the work of clinicians. Having said that, not only newer classifications but also more novel therapeutic options are required to provide more hope for OLP patients. Overall, we suggest adopting a multidisciplinary holistic approach and recommend close monitoring for these patients.
